# Human Mutations in SLC2A9 (Glut9) Affect Transport Capacity for Urate

**DOI:** 10.3389/fphys.2018.00476

**Published:** 2018-06-18

**Authors:** Anne Ruiz, Ivan Gautschi, Laurent Schild, Olivier Bonny

**Affiliations:** ^1^Department of Pharmacology and Toxicology, University of Lausanne, Lausanne, Switzerland; ^2^Service of Nephrology, Department of Medicine, Lausanne University Hospital, Lausanne, Switzerland

**Keywords:** SLC2A9, transporters, structure-function relationships, uric acid, site directed mutagenesis

## Abstract

SLC2A9 or Glut9 is a voltage sensitive urate transporter, mainly expressed in the kidneys, the liver, and the intestine. Human Glut9 loss-of-function mutations were identified in familial hypouricemia, and several single nucleotide polymorphisms (SNPs) were associated with lower serum urate, further indicating that Glut9 is a major determinant of serum uric acid level. To get insights in Glut9 transport characteristics, we systematically analyzed the function of known human Glut9 mutants using ^14^C-urate uptake assay and two-electrode voltage clamp (TEVC) in the *Xenopus laevis* oocyte expression system. Surface expression was assessed by immunostaining and biotinylation. We found decreased urate transport by flux studies for most of the variants. No variant was permissive for glucose transport. We could further differentiate two behaviors among the mutants: those harboring poor overall and cell-surface expression leading to low activity and those fully expressed at the cell surface, but presenting decreased activity. We studied the latter by TEVC and observed, in depolarized conditions, decreased inward currents measured in presence of 400 μM urate, partially reversed in 1 mM urate. In addition, we showed that C210F displays lower transport ability. By contrast, N333S showed decreased urate transport activity and urate affinity, suggesting that it may belong to the urate binding pocket. Systematic analysis of Glut9 mutants confirms Glut9 as putative target for the treatment of hyperuricemia and brings new insights in Glut9 structure – function.

## Introduction

Uric acid (UA) is a weak acid mostly found as urate in the serum at physiological pH. Serum uric acid (SUA) concentration, at steady state, is the result of a balance between its production due to the activity of the liver enzyme xanthine oxidase (Watts, 1966), and of its elimination through either its degradation by the enzyme uricase or by its excretion through the kidneys and the intestine (Sorensen, 1965). Due to successive mutations in the gene coding for uricase (Wu et al., 1992), humans lost the ability to degrade UA and have higher SUA concentrations by about thirty times compared to other species (Keilin, 1959). If this might have led to evolutionary advantages, it also contributes to significant morbidity, including gout, tophi and kidney stones.

In human kidneys, most of urate transport takes place in the proximal tubule and results in net urate reabsorption of 90–95% of the filtered load. This process is complex, with reabsorption and secretion intervening simultaneously and with many transporters involved. SLC2A9 (also called Glut9 or URATv1) proved to be central to urate reabsorption, as indicated by several lines of evidence.

First, human mutations of *SLC2A9* lead to Renal Hypouricemia type 2 (RHUC-2, OMIM #612067), a monogenic disease characterized by very low SUA (decreased by 70 to 90%), high fractional excretion of urate and occasional exercise-induced acute renal failure (Polasek et al., 2010). Several mutations in the *SLC2A9* gene were reported so far (**Table 1**) and most of them were studied functionally (Matsuo et al., 2008; Dinour et al., 2010, 2012; Stiburkova et al., 2012; Jeannin et al., 2014). However, methods and results were disparate and sometimes inconsistent, giving rise to difficulties in the interpretation of the pathogenicity of some variants. In particular, P412R, was shown to display reduced Glut9-mediated urate transport by functional studies in the *Xenopus laevis* oocytes expression system by Anzai et al. (2008), but not by Matsuo et al. (2008).

**Table 1:** List of published Glut9 variants studied in this work.

Glut9 variants	IDs	Homozygous/ heterozygous	Allele	Total allelefre quency	Origin	Biological characteristics Normal range : SUA : 155–430 μmol/L FE : 5–10%	Populations at risk (Top 3 Frequency)	Reference
L75R	rs863225072	Homozygous	A/C	1.682^∗^10^-5^	Human	SUA = 10 μmol/L FE>150%	European (4.232^∗^10^-5^)	Dinour et al., 2010
T125M	rs181509591	Homozygous	G/A	1.263^∗^10^-4^	Human	SUA = 12 μmol/L FE>150%	Latino (6.974^∗^10^-4^), Other (1.54^∗^10^-4^), European (7.9^∗^10^-5^)	Dinour et al., 2012
R171C	rs776127501	Heterozygous Homozygous	G/A	4.069^∗^10^-5^	Human	SUA = 259 μmol/L FE = 4.9% SUA = 6 μmol/L FE = 128%	South Asian (9.746^∗^10^-5^), East Asian (5.795^∗^10^-5^), European (5.393^∗^10^-5^)	Dinour et al., 2012
R198C	rs121908322	Heterozygous	G/A	3.262^∗^10^-5^	Human	SUA = 125 μmol/L	South Asian (1.3^∗^10^-4^), Latino (2.98^∗^10^-5^), European (2.71^∗^10^-5^)	Matsuo et al., 2008
G216R	rs561633150	Homozygous	C/T	5.607^∗^10^-4^	Human	(Associated with N333S)SUA = 40 μmol/L FE = 93%	South Asian (4.971^∗^10^-3^), Other (3.1^∗^10^-4^)	Stiburkova et al., 2012
N333S	rs75348295	Heterozygous	T/C	8.176^∗^10^-6^	Human	(Associated with G216R heterozygous)SUA = 30 μmol/L FE = 46%	European (1.811^∗^10^-5^)	Stiburkova et al., 2012
R380W	rs121908321	Heterozygous	G/A	1.986^∗^10^-4^	Human	SUA = 90 μmol/L FE = 16%	South Asian (1.3^∗^10^-3^), East Asian (3.712^∗^10^-4^), Latino (8.718^∗^10^-5^)	Matsuo et al., 2008
P412R	rs121908323	Heterozygous	G/C	5.98^∗^10^-6^	Human	SUA = 143 μmol/L	East Asian (8.295^∗^10^-5^)	Anzai et al., 2008; Matsuo et al., 2008
C210F	n.a				Dog			Bannasch et al., 2008
V253I	rs16890979		C/T	0.245	Human	SUA -26.17 ± 3.57 μmol/L	African (0.436), Latino (0.3978), South Asian (0.2428)	McArdle et al., 2008; Dehghan et al., 2008
R265H	rs3733591		C/T	0.239	Human	SUA -5.95 ± 4.16 μmol/L	East Asian (0.668), South Asian (0.3619), European (0.2389)	McArdle et al., 2008; Yang et al., 2010; Hollis-Moffatt et al., 2011
P350L	rs2280205		G/A	0.437	Human	SUA -7.14 ± 29.74 μmol/L	Ashkenazi Jewish (0.563), Finnish European (0.5403), Other European (0.5182)	McArdle et al., 2008; Stiburkova et al., 2012

Second, if Glut9 mutants associated with RHUC-2 are rare and occur in less than 1% of the population, some frequent single nucleotide polymorphisms of *SLC2A9* (V253I, rs16890979; R265H, rs3733591; and P350L, rs2280205) were associated with slightly lower SUA reduction of 5 to 10% (McArdle et al., 2008; Hurba et al., 2014).

Third, two animal models carrying defective Glut9 proteins (Dalmatian dog and mouse) displayed increased fractional excretion of urate. Indeed, genetic analysis of the hyperuricosuric Dalmatian dog breed revealed the presence of the C188F mutation (corresponding to residue C210 in humans) in the canine SLC2A9 gene (Bannasch et al., 2008) and Glut9 knockout mice display high fractional excretion of urate, leading to hyperuricosuria, tubular obstruction and renal failure (Preitner et al., 2009).

Finally, Anzai et al. (2008), Caulfield et al. (2008), Vitart et al. (2008), Bibert et al. (2009) showed that Glut9 transports urate efficiently with a *K*_m_ of ∼650 μM and could be modulated by the presence of fructose (Long et al., 2015). Glut9 bears all characteristics of the basolateral voltage-sensitive renal urate transporter previously described by Roch-Ramel and Guisan (1999; Li et al., 2007; Dehghan et al., 2008; Yang et al., 2010; Sulem et al., 2011). In particular, Glut9-mediated urate transport proved to be sodium- and chloride-independent, voltage-sensitive, and electrogenic (Anzai et al., 2008; Caulfield et al., 2008; Vitart et al., 2008; Bibert et al., 2009). Of note, two isoforms of hGlut9 are known: a long isoform (Glut9a, 12 exons, 540 amino acids) and a short isoform (Glut9b or Glut9ΔN, 13 exons, 512 amino acids), that differ only at their N-terminal part and display slight functional differences (Anzai et al., 2008; Caulfield et al., 2008; Bibert et al., 2009).

Secondary structure of the Glut family was previously described in great details for Glut1 by site-directed mutagenesis. It was demonstrated that the protein harbors 12 transmembrane domains, intracytoplasmic NH_3_- and COOH-terminal parts and a single *N*-glycosylation site in the first extracellular loop (Mueckler et al., 1985). These findings were recently confirmed and refined by the resolution of the crystals of the closest bacterial homolog, the xylose-H^+^ transporter *XylE* and by the crystals of human Glut1 and Glut5 (Sun et al., 2012; Deng et al., 2014; Nomura et al., 2015). In absence of mammalian Glut9 crystal, we recently built up a first homology model based on *XylE*’s crystal coordinates (Clemencon et al., 2014) and others have recently studied critical Glut9 residues based on an homology model developed on Glut5 crystal structure (Long et al., 2017). While these major structural achievements helped understanding Glut9 architecture, several questions remain open, including the precise localization of the binding site for urate or the urate translocation mechanisms. In this study, we took advantage of known human and Dalmatian loss-of-function mutants to systematically explore the expression and activity of Glut9 and get insights about its structure.

## Experimental Procedures

### Materials and Chemicals

Mouse GLUT9a and b and GLUT2 complementary DNAs (cDNAs) were obtained as described previously (Bibert et al., 2009). Human GLUT9a and b cDNAs were purchased from Imagene and subcloned in pSDeasy or in triple-FLAG (3xF)-containing pCMV expression vectors. Amino acid numbering refers to human Glut9a throughout the manuscript.

[^14^C]-urate with an activity of 50–60 mCi/mmol and [^14^C] D-glucose with an activity of 2–10 mCi/mmol were purchased from American Radiolabeled Chemicals, Inc.

### Site-Directed Mutagenesis

Mutants were generated using two-steps PCR, as described (Bonny et al., 1999). Primers were obtained from Microsynth AG, Balgach, Switzerland. Amplicons and pSDeasy plasmid vectors containing hGLUT9 were digested with cassettes as described in **Table 2**. All constructs have been verified by sequencing (Synergene Biotech GmbH, Zurich, Switzerland).

**Table 2:** List of primers and cassette used for mutagenesis.

Cassette	Flanking primers	Mutation	Forward primer	Reverse primer	Restriction enzyme
	4920		5′ACGCGGCTACAATTAATACATAACC3′		
	893			5′TCGTTGTGCTTCTCCAAGAGCAGG3′	
		L75R	5′TACGGCTACAACC**ga**TCGGTGGTGAATGCCCC3′	5′GGGGCATTCACCACCGA**tc**GGTTGTAGCCGTA3′	
		T125M	5′GGACTTGTGGGGA**g**ATTAATTGTGAAGATGATTGG3′	5′CCAATCATCTTCACAATTAAT**c**TCCCCACAAGTCC3′	
I		R171C	5′CTCATCGTGGGA**t**GCTTCATCATG3′	5′CATGATGAAGC**a**TCCCACGATGAG3′	*EcoR*I-*Blp*I
		R198C	5′CCCAAGGAGATC**t**GTGGCTCTCTGGG3′	5′CCCAGAGAGCCAC**a**GATCTCCTTGGG3′	
		C210F	5′ACTGCCATCTTTATCT**t**CATTGGCGTGTTCAC3′	5′GTGAACACGCCAATG**a**AGATAAAGATGGCAGT3′	
		G216R	5′GGCGTGTTCACT**c**GGCAGCTTCTG3′	5′CAGAAGCTGCC**g**AGTGAACACGCC3′	
	766		5′CTGCTGGGAAAGGAGAGTACCTGG3′		
	1415			5′TCACCAGTCAAGATGAACGGGATGC3′	
II		V253I	5′AAAGCAGAC**a**TTTCCCAAGAGGTAGAGGT3′	5′ACCTCTACCTCTTGGGAAA**t**GTCTGCTTT3′	*Blp*I-*Sbf*I
		R265H	5′CTGGCTGAGAGCC**a**CGTGCAGAGG3′	5′CCTCTGCACG**t**GGCTCTCAGCCAG3′	
	1197		5′ACCCCTCCTCATTGGTGGCTTTGG3′		
	2760			5′AGGGGTCACAGGGATGCCACCC3′	
III		P350L	5′AAAGCTGGGATCCCTC**c**GGCAAAGATCCC3′	5′GGGATCTTTGCC**g**GAGGGATCCCAGCTTT3v	*Sbf*I-*Xho*I
		R380W	5′ATTGAGCACCTGGGA**t**GGAGACCCCTCCT3′	5′AGGAGGGGTCTCC**a**TCCCAGGTGCTCAAT3′	
		P412R	5′CTGCAGGACCACGCCC**g**CTGGGTCCCCTA3′	5′TAGGGGACCCAG**c**GGGCGTGGTGGTCCTGCAG3′	

*Lower case and bold letters indicate the mutations introduced in the sequence.*

### *In Vitro* Transcription

All mutants and wild-type cDNAs were linearized by *Pvu*I (Promega), except for L75R where *Nhe*I (Invitrogen) was used. *In vitro* transcription was performed with SP6 mMESSAGE mMACHINE Kit (Ambion, Life Technology). The cRNAs were purified using RNeasy Mini Kit (QIAGEN).

### Expression Into *X. laevis* Oocytes

Stages V–VI oocytes were selected from *X. laevis* (African Xenopus Facility C.C, Knysna, Republic of South Africa and Xenopus Express, Le Bourg, France). Experiments were performed with the authorization of the Veterinarian Office of the Canton de Vaud. Oocytes were injected with 10 ng of either wild-type or mutant cRNAs, or equivalent volume of water and incubated 48 h in Modified Barth’s Solution (MBS) before experiments were performed.

### Western Immunoblotting

Ten oocytes of each condition were prepared as described (Geering et al., 1996). Two μg of proteins were supplemented with 2X solubilization buffer (SB) (60 mM Tris pH6.8, 5% SDS, 20% Glycerol and 0.2% Bromophenol blue) containing 1.5% of 2-mercaptoethanol and then heated 10 min at 56°C. Proteins were separated on a 10% SDS polyacrylamide gel and transferred onto nitrocellulose membrane (GE Healthcare Life Science). Membranes were incubated overnight with anti-mouse FLAG monoclonal antibody (Sigma) (1:500), followed by 1 h with anti-mouse IgG-HRP (GE Healthcare Life Science), revealed by ECL (Pierce Biotechnology, Inc.) and exposed on Kodak films.

### Biotinylation

All biotinylation steps were performed at 4°C and according to Harris et al. (2007). Twenty oocytes per condition were placed for 15 min in biotinylation buffer containing 1 mg/mL EZ-link sulfo-NHS-SS-Biotin (Pierce Biotechnology, Inc.), 10 mM Triethanolamine, 150 mM NaCl, 2 mM CaCl_2_pH9.5. Quench buffer (192 mM Glycine, 25 mM Tris HCl pH7.5 in MBS) was added to stop the reaction, before washing three times with MBS. Oocytes were then lysed [1% Triton X-100, 100 mM NaCl, 5 mM EDTA, 50 mM Tris HCl pH 7.5, 0.5% Na deoxycholate and protease inhibitors (cOmplete Protease Inhibitor Cocktail Tablet, Roche) and centrifuged 10 min at 12’000 rpm]. The intermediate phase was transferred into new tubes and was exposed overnight to NeutrAvidin Agarose Resin beads (Pierce Biotechnology, Inc.). Beads were washed three times with lysis buffer and heated 10 min at 56°C in SB. Western blot was performed as described above. Four biotinylation assays were performed on independent batches of oocytes. Results were quantified using Image J.

### Urate and Glucose Uptake in *X. laevis* Oocytes

Oocytes were incubated 45 min in MBS containing either 100 μM [^14^C]-urate with 300 μM cold urate (Bibert et al., 2009) for urate uptake or containing 1 mM [^14^C] D-glucose for glucose transport studies. Oocytes were washed five times in cold MBS and transferred individually into vials containing 100 μL 5%SDS. Two mL of scintillation liquid (PerkinElmer) was added before counting in a Packard analyzer. Three independent batches with 10 oocytes per condition were pooled.

### Two Electrodes Voltage Clamp in *X. laevis* Oocytes

Measurements were performed using a TEV-200A electrode clamp (Dagan) and measured with Digidata (Axon instruments). Oocytes were clamped at 0 mV and the current was recorded for 20 s in presence or absence of different concentrations of urate in 100 mM NaCl, 2.5 mM KCl, 10 mM Na-HEPES, 1 mM MgCl_2_, 0.8 mM CaCl_2_ kept at pH 7.4. For I/V measurements, recordings were performed from -120 mV to +60 mV with 20 mV increment steps. Each experiment was performed on three independent batches with atleast five oocytes per batch.

Urate dose/response studies were performed by measuring sequentially the current after the perfusion of increasing concentrations of urate for 30 s (0.4, 1, 2, 3, and 5 mM). The voltage was clamped at 0 mV and each experiment was done on five oocytes per condition and per batch (three batches).

### Immunohistochemistry

Eight μm oocyte cryosections were washed in PBS, blocked in Normal Goat Serum (NGS) blocking buffer (10%NGS, PBS 1X, 0.5% Tween in water) and incubated in the primary mouse anti-FLAG antibody (Sigma) (1:500). Endogenous peroxidase was quenched using 0.3% H_2_O_2_ (Sigma-Aldrich) and secondary biotinylated anti-mouse antibody (1:200) (Vector Laboratories) was added. The slides were washed and treated for 30 min with the avidin-biotin complex (ABC) horseradish peroxidase (HRP). VECTOR NovaRED Peroxidase Substrate kit (Vector Laboratories) was used for detection. Sections were visualized under EVOSfl microscope (AxonLab).

### Building of the Glut9 3D Homology Model

3D model of human Glut9 was built by homology with the human Glut1 transporter (pdb: 4PYP), using MODELLER 9.1 (Webb and Sali, 2014). View of the model was prepared with UCSF Chimera (Pettersen et al., 2004). Full fitness was -1756,66 kcal⋅mol^-1^ and ΔG = -7.1 kcal⋅mol^-1^.

### Statistics

Statistics were performed using Graphpad Prism 6.01 (Graphpad Software Inc.). Kruskal–Wallis test followed by a Dunnett’s correction test was used to compare mutants to wild-type in the ^14^C uptake assay. Multiple *T*-tests followed by the Bonferroni correction were used to compare protein expression with the activity displayed by every mutant. Finally, a Dunnett’s multiple comparison test was used to compare concentration-response of urate in mutant and water injected oocytes vs. WT. *V*_max_ and *K*_m_ of urate kinetic analysis were determined from a non-linear regression. *P* was considered as significant if <0.05.

## Results

### Transport Activity and Expression of Human Glut9 Mutants

**FIGURE 1:**
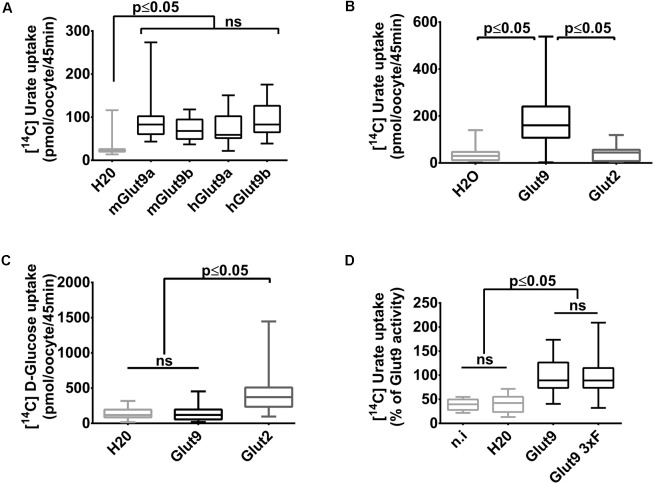
Glut9-mediated [^14^C] urate uptake in *Xenopus laevis* oocytes. **(A)** [^14^C] urate uptake was measured after 45 min incubation time in *X. laevis* oocytes injected with human or mouse Glut9a, Glut9b or with water (H_2_O). Total concentration of urate in the uptake solution is 400 μM. **(B,C)** Glut9 transports [^14^C] urate, but not [^14^C] D-Glucose. **(D)** The introduction of a triple flagged tag (Glut9 3xF) did not affect urate transport by Glut9. Statistical analysis were performed using a Kruskal–Wallis test followed by a correction with Dunnett’s test (*p* < 0.05). Each bar represents the mean and standard deviation of 3 independent batches of oocytes, with 10 oocytes per batch and condition. N.i, not injected; NS, not significant.

We have previously shown that mouse Glut9 (mGlut9) transports urate and we have suggested that the simplest model of transport fitting our observations was a uniporter (Bibert et al., 2009). Here, we first assessed human and mouse Glut9 isoforms a and b, by [^14^C] urate uptake into *X. laevis* oocytes and showed that they display the same transport rate (**Figure 1A**).We further verified that urate transport was specific for Glut9 by comparing urate and glucose uptake using Glut2 as control (**Figures 1B,C**). Finally, and for convenience, a triple FLAG-tag epitope was added to the C-terminal part of Glut9, with no effect on urate uptake (**Figure 1D**).

**FIGURE 2:**
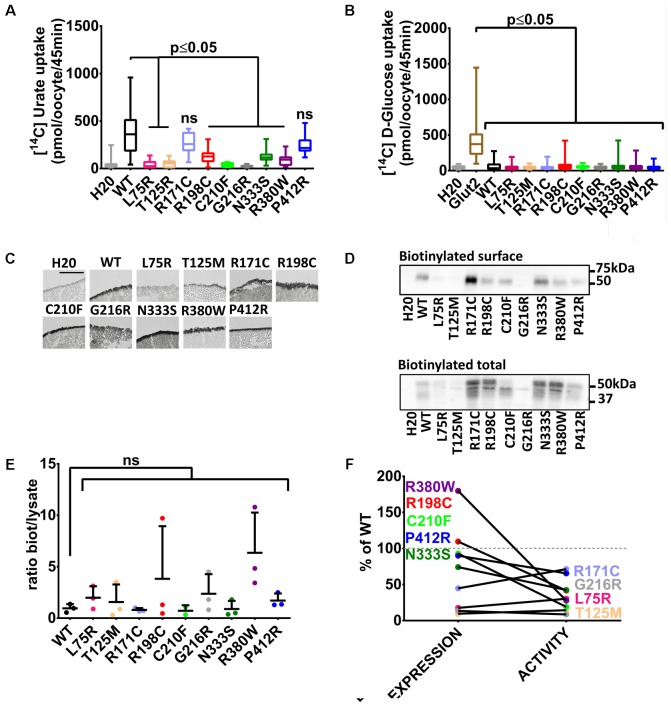
Transport studies on human Glut9 loss-of-function mutations. **(A)** [^14^C] urate uptake assay was systematically performed on published Glut9 loss-of-function mutants and revealed decreased activity for all of them (i.e., L75R, T125R, R198C, C210F, G216R, N333S, and R380W) except for mutants R171C and P412R that do not reach significance. **(B)** None of these mutants displayed D-glucose transport activity when tested for [^14^C] D-glucose uptake. **(C)** Immunohistochemistry with an anti-Flag antibody on cryosections of oocytes injected with WT-Glut9 or mutant showed conserved expression at the cell surface for R171C, R198C, C210F, N333S, R380W, P412R and a decreased cell surface expression for L75R, T125M, and G216R. Representative pictures of three experiments from different oocyte batches are shown. **(D)** Cell surface assessment by biotinylation assay on oocytes injected either with Glut9 WT or the designated Glut9 mutants. One representative experiment out of 4 is shown. **(E)** Densitometric analysis revealed no difference in the ratio between biotinylation and total lysates for each Glut9 mutant when compared to WT Glut9. **(F)** Summary of cell surface expression (biotinylation) and transport activity (urate uptake) of all tested mutants reported to Glut9 WT. R198C, C210F, N333S, R380W, and P412R (in black) are the Glut9 mutants displaying preserved cell surface expression but decreased transport activity. L75R T125M, R171C, and G216R (in gray) are less expressed than WT and except for R171C, have less activity. Statistical analysis was performed using a Kruskal–Wallis test followed by a correction with Dunnett’s test (*p* < 0.05). Each experiment was performed on 3 independent batches of oocytes with 10 oocytes per batch and condition for uptake assay and 20 oocytes per condition for the biotinylation assay.

With these tools in hands, Glut9 human and dog loss-of-function mutations published so far (see **Table 1**) were systematically examined in order to determine the cause of the decreased activity *in vivo* and gain insights in Glut9 structure/function. Most of the mutants (L75R, T125M, R198C, C210F, G216R, N333S, and R380W) displayed significant decreased [^14^C]-urate uptake, while R171C and P412R had preserved [^14^C]-urate uptake (**Figure 2A**), with non-significant lower uptake compared to wild-type Glut9 and significantly higher uptake than water-injected oocytes. None of the Glut9 mutations showed any [^14^C]-glucose uptake (**Figure 2B**), indicating that the mutations did not result in a loss of affinity for urate in favor of glucose.

We studied the expression level of Glut9-mutants compared to wild-type Glut9-injected oocytes. Cell surface analysis by immunohistochemistry revealed a strong decrease of protein expression for Glut9 carrying the mutations L75R, T125M, and G216R, while other mutants were expressed at a comparable level as compared to wild-type Glut9 (**Figure 2C**). These findings were further analyzed and quantified by a surface biotinylation assay (**Figure 2D**). Interestingly, T125M and G216R appeared to be expressed only in the non-glycosylated form, while R171C expression seemed stronger in the fully glycosylated state. However, when we normalized the cell surface expression (biotinylated fraction) to the overall cell expression (total lysate), no difference was observed between wild-type Glut9 compared to the mutants (**Figure 2E**), suggesting that the decreased surface expression was not due to defective sorting of the protein, but mainly to decreased expression. **Figure 2F** summarizes the expression level (surface biotinylation) and the transport activity (urate uptake) of each of the mutant studied and allows separation in two main categories: a group of mutants which are well expressed and are present at the cell surface (R171C, R198C, C210F, N333S, R380W, and P412R) and a group of mutants less well expressed and having low transport activity (L75R, T125M, and G216R).

### Transport Activity and Expression of Human Glut9 Single Nucleotide Polymorphisms (SNPs)

**FIGURE 3:**
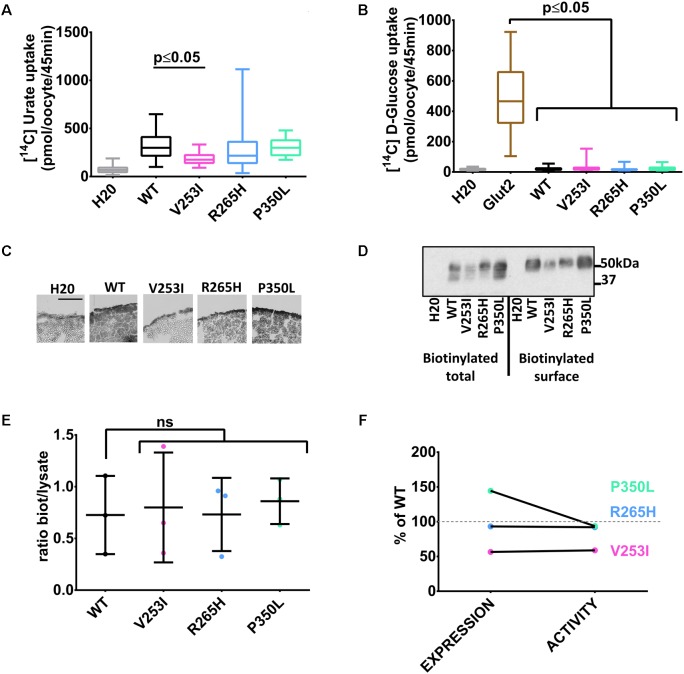
Transport studies on Glut9 SNPs associated with low SUA. [^14^C]urate **(A)** and [^14^C]glucose **(B)** uptake in oocytes injected with Glut9 SNPs. **(C)** Immunohistochemistry with an anti-Flag antibody of cryosections of oocytes injected with WT-Glut9 and SNPs V253I, R265H, and P350L reveals a decreased expression at the cell surface for V253I. Protein expression was also verified by biotinylation and densitometric analysis **(D,E). (F)** Comparison of expression level and activity of WT-Glut9 or SNPs. Statistical analysis was performed using a Kruskal–Wallis test followed by a correction with Dunnett’s test (*p* < 0.05) with 3 batches of oocytes with 10 oocytes per batch and per condition for the fluxes and 20 oocytes for biotinylation.

Three human Glut9 SNPs (V253I, R265H, and P350L) were associated with low SUA in a genetic association study (**Table 1**) (McArdle et al., 2008). SNPs transport activity was tested in the *X. laevis* oocyte expression system. [^14^C]-urate transport was significantly lower for the variant V253I when compared to the wild-type (**Figure 3A**). No transport of [^14^C]-glucose was observed (**Figure 3B**). Cell surface expression assessed by immunohistochemistry showed decreased expression for variant V253I (**Figure 3C**). This decrease was confirmed by the biotinylation assay in which the expression of V253I was decreased both in total lysate and at the cell surface (**Figure 3D**). The ratio between total protein expression and cell surface expression was constant, suggesting that no main trafficking issue was accounting for the decreased expression (**Figure 3E**). This data indicates that the SNP V253I has low urate transport activity due to lower expression (**Figure 3F**). The two other SNPs studied here were not different from wild-type when expressed in the oocyte expression system.

### Electrophysiological Studies of Glut9 WT and Variants

**FIGURE 4:**
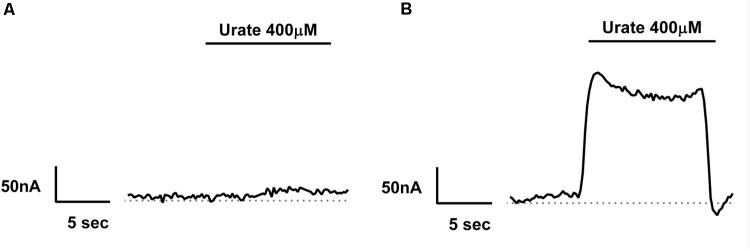
Electrogenicity of human Glut9 upon urate exposure. Representative trace current of H_2_O-**(A)** or Glut9-**(B)** injected oocytes in presence of 400 μM urate. The dashed line represents the current at baseline (0 mV).

**FIGURE 5:**
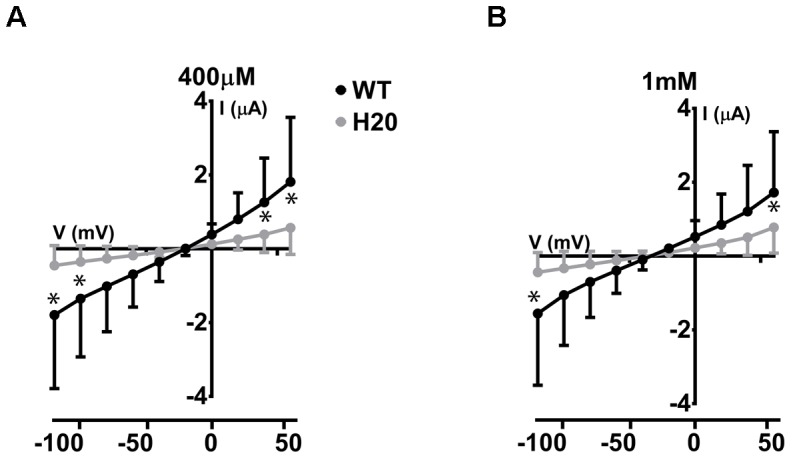
Electrophysiological studies of WT-Glut9. I/V curves for H_2_O-injected and WT-Glut9-injected oocytes exposed to 400 μM **(A)** or 1 mM **(B)** of urate. Three batches of oocytes were used with 5 oocytes per batch. Data are means ±SD. Asterisks denote *p* < 0.05 between Glut9- and H_2_O-injected oocytes.

We have previously described electrogenic urate transport in *X. laevis* oocytes injected with mouse Glut9 cRNA (Bibert et al., 2009). Whether human Glut9 displays the same characteristics was assessed by two-electrode voltage clamp at 0 mV measured for 30 s in presence or not of 400 μM urate. We observed positive currents in the 100 nA range upon application of urate (**Figures 4A,B**). We further applied a voltage-clamped protocol with voltage increment of 20 mV from -120 mV to +60 mV in presence of 400 μM or 1 mM urate and measured the currents. I/V curves were established. The negative reverse potential is compatible with the transport of anions. For WT Glut9, no significant difference in I/V curves could be made between the two extracellular urate concentrations tested (400 μM and 1 mM). H_2_0-injected oocytes had small currents influenced by the voltage and by extracellular urate concentration, probably due to some endogenous transporters present in the oocyte (**Figures 5A,B**).

**FIGURE 6:**
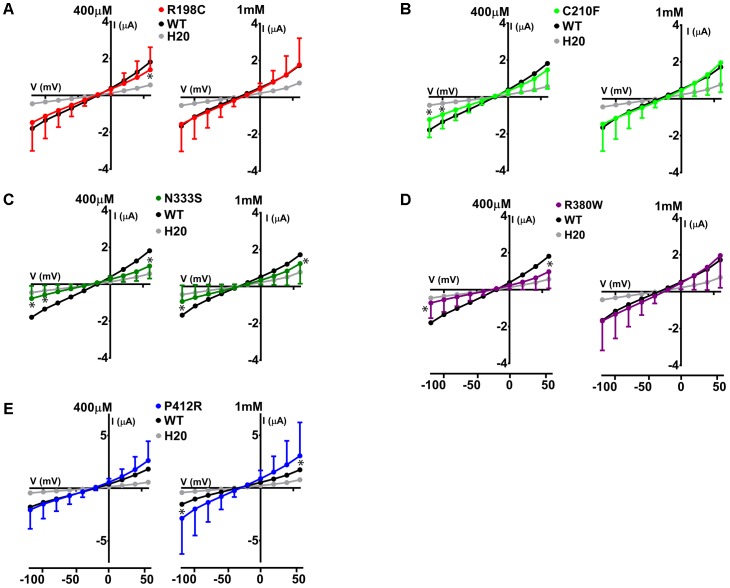
Electrophysiological studies of mutants Glut9. I/V curves for the following Glut9 mutants measured at 400 μM and 1 mM urate were built: R198C **(A)**, C210F **(B)**, N333S **(C)**, R380W **(D)**, and P412R **(E)**. For the ease of comparison, the I/V curves for Glut9-WT and for water-injected-oocytes are represented on each panel. Three batches of oocytes were used with 5 oocytes per batch. Asterisks denote *p* < 0.05 by unpaired *t*-test between WT-injected and mutant-injected oocytes.

**FIGURE 7:**
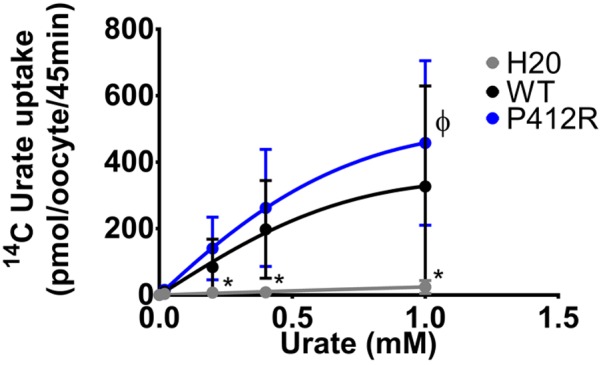
Concentration-response analysis for P412R. [^14^C]urate uptake was measured in H_2_O, WT, or P412R-injected oocytes at increasing concentrations of urate: 0.02, 0.20, 0.40, and 1 mM. Ten oocytes per batch on 3 batches of oocytes were used. A Dunnett’s multiple comparison test was used to compare P412R to WT and water (^∗^*p* < 0.05).

The same protocol was used to study the mutants showing an expression level comparable to WT Glut9, but having low activity by [^14^C]-urate uptake (R198C, C210F, N333S, R380W, and P412R). R198C, C210F, and R380W had currents lower than WT-Glut9 in presence of 400 μM of urate, a difference that disappeared at higher extracellular urate concentration (1 mM) when the membrane was highly depolarized (above +20 mV) (**Figures 6A,B,D**). N333S displayed also lower currents than WT-Glut9, but the increase in current after exposure to 1 mM urate does not allow full recovery (**Figure 6C**). Finally, P412R had higher currents than WT-Glut9 in the depolarized state in presence of 400 μM of extracellular urate, getting even higher at 1 mM urate (**Figure 6E**). This interesting increase of activity of Glut9 carrying the mutation P412R prompted us to verify [^14^C]-urate uptake at increasing concentrations of urate (**Figure 7**). We found higher urate uptake in presence of higher extracellular urate concentrations.

**FIGURE 8:**
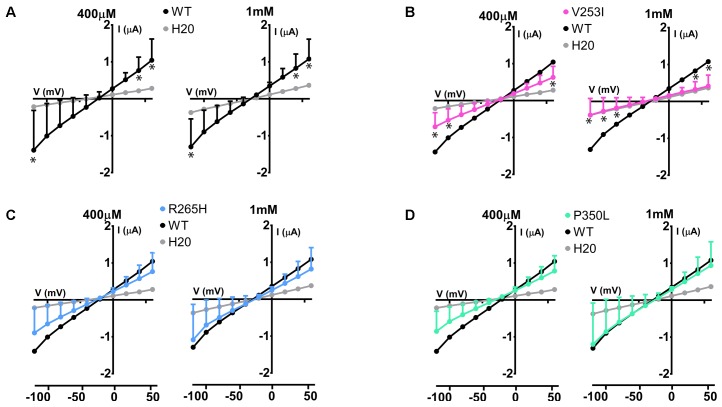
Electrophysiological studies of Glut9 SNPs. I/V curves built upon exposure to 400 μM or 1 mM of urate for: Glut9 WT and water-injected oocytes **(A)** or Glut9 SNPs injected-oocytes: V253I **(B)**, R265H **(C)**, and P350L **(D)**. For the ease of comparison, the I/V curves for Glut9-WT and for water-injected-oocytes are represented on each panel. Three different batches of oocytes were used with 5 oocytes per batch. ^∗^Represents *p* < 0.05 compared to WT-injected oocytes.

The same study was performed on Glut9 SNPs V253I, R265H, and P350L. V253I showed a decrease of the currents in presence of 400 μM of urate, further decreased at 1 mM urate (**Figure 8B**). R265H and P350L showed only minimal changes in currents compared to WT-Glut9, whether the concentration of urate perfused is 400 μM or 1 mM (**Figures 8A,C,D**).

**FIGURE 9:**
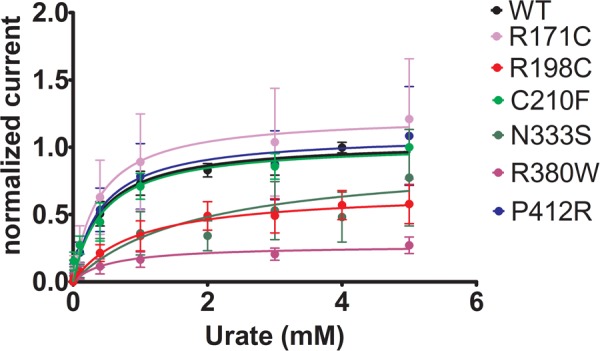
Urate kinetics of WT-Glut9. Glut9-injected oocytes were sequentially exposed to increasing urate concentrations (0.01, 0.1, 0.4, 1, 3, and 5 mM) and current was recorded by TEVC, clamped at 0 mV. Each measurement was repeated on atleast 5 oocytes per batch on atleast 3 batches. Detail analysis of *V*_max_ and *K*_m_ are reported in **Table 3**.

To further study the electrophysiological characteristics of Glut9 and the impact of the mutations on its properties, we looked at the current response to increasing concentrations of urate (0.01, 0.1, 0.4, 1, 3, and 5 mM) at a fixed voltage (0 mV) and fitted the values according to Michaelis–Menten kinetics. Urate kinetics for Glut9-WT revealed a *V*_max_ ≈ 347 nA with a *K*_m_ of ≈0.43 mM (**Figure 9**). The same protocol was used for each expressed mutant and the results obtained are summarized in **Table 3**. Most mutants showed decreased *V*_max_ compared to Glut9-WT, except R171C, N333S, and P412R. All mutants but N333S had the same affinity for urate as Glut9-WT, with the exception of N333S that displayed higher *K*_m_ than WT, indicative of lower affinity for substrate (**Table 3**).

**Table 3:** Urate kinetics measurements of Glut9 mutants.

	*V*_max_ (μA) (mean ±*SD*)	*K*_m_ (mM) (mean ±*SD*)
WT	0.347 ± 0.175	0.433 ± 0.197
R171C	0.345 ± 0.105	0.411 ± 0.119
R198C	0.181* ± 0.056	0.603 ± 0.313
C210F	0.158* ± 0.056	0.497 ± 0.299
N333S	0.260 ± 0.169	1.221* ± 0.799
R380W	0.076* ± 0.021	0.839 ± 0.731
P412R	0.313 ± 0.082	0.434 ± 0.960

*Oocytes were exposed to increasing extracellular urate concentrations (0.01, 0.1, 0.4, 1, 3, and 5 mM) and currents were measured by TEVC. The voltage was clamped at 0 mV. V_max_ and K_m_ were determined from a non-linear regression. Each value represents the mean of atleast 5 oocytes per batch from atleast 3 batches. SD, standard deviation. ^∗^p < 0.05 compared to WT by 1 way ANOVA.*

## Discussion

This study identified two main behaviors for Glut9 mutants and variants: either the mutants were poorly expressed in the oocyte expression system, leading to decreased transport activity, or the mutants had preserved protein expression, and the activity was decreased through changes in the intrinsic transport activity. Both resulted in lower urate transport ability and explain the loss-of-function phenotype encountered in hypouricemic patients carrying these mutations, except for P412R and R171C which display only a moderate decrease in [^14^C]-urate transport ability.

**FIGURE 10:**
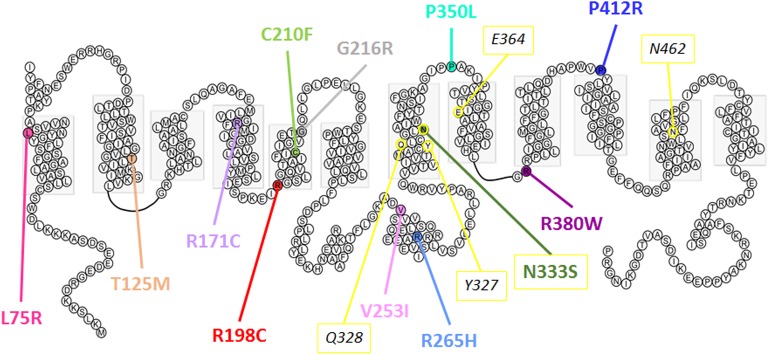
Predicted secondary structure of Glut9. Representation of human Glut9 mutations and SNPs associated with variations of serum uric acid. Transmembrane domains are indicated by gray boxes. Extracellular side is upper and intracellular side is lower. The corresponding five main residues involved in glucose binding in hGLUT1 have been highlighted in yellow as indication.

In the oocyte expression system, decreased expression level of mutants L75R, T125M, and G216R was probably not due to disturbed trafficking of the protein to the cell membrane, but instead was due to a general decreased expression. RNA decay or early protein degradation after quality check could account for it. These mechanisms were, however, not further studied here. Of note, mutations leading to poor expression are mainly found in the transmembrane domains and are predicted to disrupt the overall structure of the protein (**Figure 10**).

The second category of mutants (R171C, R198C, C210F, N333S, R380W, and P412R) provides valuable information on the mechanisms of urate transport through Glut9. If they were all well expressed at the cell surface, their activity was decreased significantly when studied by urate fluxes and by electrophysiology. Interestingly, in electrophysiological studies, the measured current, especially at high voltage, could be rescued when higher extracellular urate concentrations were applied. Further insights are provided by the analysis of the position of these mutants in the protein. They are all comprised in the transmembrane domains previously identified by site-directed mutagenesis as important for Glut1-mediated glucose transport and probably forming the glucose binding pocket in Glut1 (**Figure 10**).

**FIGURE 11:**
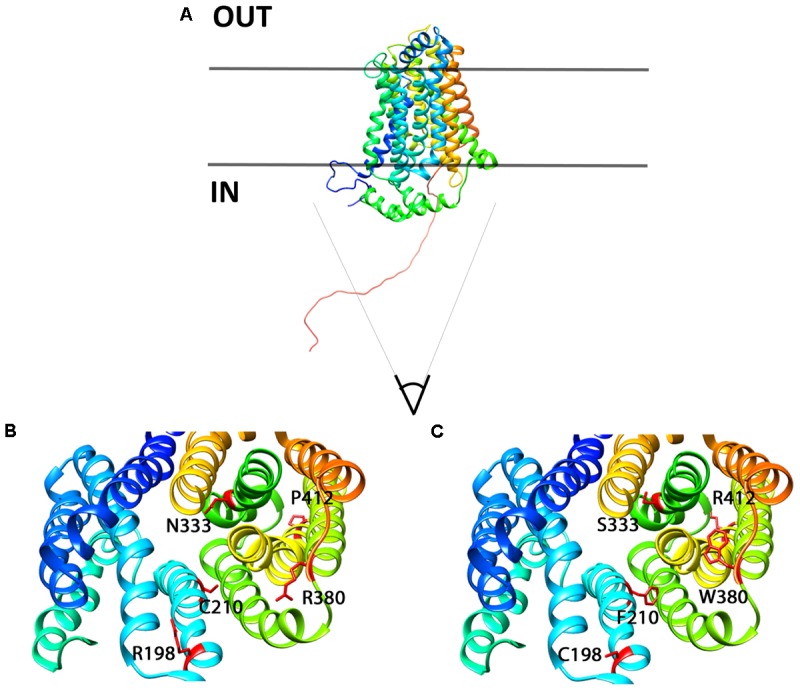
3D homology model for Glut9, based on human Glut1 crystal structure. **(A)** Lateral view of Glut9-WT. **(B,C)** Represent views from the cytoplasmic side into the direction of the putative pore of the transporter. The different helices are illustrated and the residues involved in human familial hypouricemia are illustrated in red, as WT amino acids on **(B)** and as mutated amino acids on **(C)**.

In order to better localize the precise position of the mutants, we designed a homology 3D model of Glut9 based on the human Glut1 crystal recently published (Deng et al., 2014) (**Figure 11**). We chose Glut1 and not the more recently described Glut5 structure for two main reasons. First, the two proteins share strong identity and similarity. Second, several amino acids mutated in the human gene coding for Glut9 (R171, R198, C210, and R380) and leading to familial hypouricemia type 2 are also mutated in the human Glut1 gene (R126, R153, V165, R333) and cause Glut1 deficiency syndrome, also called De Vivo syndrome (Pascual et al., 2004). We found that all the mutants of the second category (well expressed, with decreased function) are present along the putative pore of Glut9, facing the internal lumen. Further projections show that mutated residues are predicted to either protrude in the lumen of the pore or interact physically with some of the neighboring loops, suggesting that they might induce significant changes in the structure of the protein.

C210F deserves a special note. This loss-of-function mutant described in the Dalmatian dog breed, but never functionally studied, was shown to be well expressed, but had only marginal residual activity. Kinetic analysis revealed reduced transport capacity but similar affinity for urate as compared to Glut9-WT. This residue stands in the 5th transmembrane domain, facing the pore and facilitating urate transport. By converting a polar residue into an hydrophobic one, C210F probably decreases the activity without altering the overall shape and thus expression of the protein (Braun and von Heijne, 1999).

P412R turned to be intriguing. Initially identified in a patient with a mild phenotype and carrying P412R in the heterozygous state (Anzai et al., 2008), its causative role in hypouricemia was challenged by expression studies which were highly contradictory, with decreased activity (Anzai et al., 2008) or no effect (Matsuo et al., 2008) described thus far. In accordance with Matsuo et al. (2008) we found no significant alteration of urate uptake in oocytes injected with P412R in presence of 400 μM of urate. By contrast, when challenged with higher external urate concentration (up to 1 mM) we observed an increased transport activity for both, uptake and current (**Figures 6E, 7**), with no change in affinity, though. This is reminiscent to what was found for some Glut1 residues close to the equivalent position in transmembrane domain 10 and displaying increased activity for both, uptake and current (**Figures 6E, 7**), with no change in affinity, though. This is reminiscent to what was found for some Glut1 residues close to the equivalent position in transmembrane domain 10 and displaying increased activity when mutated in cysteine (I369, V370, I372, and A377) (Mueckler and Makepeace, 2002). Thus, the role of P412R remains ambiguous and no definitive conclusion can be drawn from our studies performed in the *X. laevis* oocyte expression system.

A key finding of this work is the lower affinity for urate of the mutant N333S. N333 occupies a critical position in the pore and its change in a serine decreased significantly Glut9 a ffinity for urate. This was illustrated by an increased *K*_m_ corroborated by the fact that even 1 mM urate was not able to restore full activity. This indicates that N333 is either situated directly in the urate binding pocket or that N333S influences directly urate binding. Interestingly, the key role of N333 in the transmembrane translocation of Glut9 substrates was recently illustrated in an analogy model of Glut9 built on the newly-deciphered Glut5 crystal structure (Long et al., 2017).

The functional analysis performed here on Glut9 single nucleotide polymorphisms that are associated with lower SUA and that were previously identified in a cohort of Old Order Amish (McArdle et al., 2008) showed that one SNP (V253I) is less expressed at the cell membrane and has less transport activity than WT-Glut9. This finding was further confirmed by electrophysiological measurements showing that V253I is particularly sensitive to high extracellular urate concentrations. These results are in slight contradiction with Hurba et al. (2014) who showed a trend toward lower Glut9 activity for this variant, not reaching significance though (Hurba et al., 2014). We provide here strong evidence that surface expression (by two methods: biotinylation and immunofluorescence) and activity (by urate uptake and by TEVC) are decreased for this SNP in the heterologous expression system of the *X. laevis* oocyte. The two other SNPs studied did not behave differently compared to WT. Further studies in human will be necessary in order to show the relevance of these SNPs on urate homeostasis in larger populations (Mancikova et al., 2016).

## Conclusion

Overall, our study reveals that some loss-of-function Glut9 mutants are in direct interaction with the binding pocket and the pore of the Glut9 urate transporter. We also showed for the first time that the Dalmatian mutation C210F decreased Glut9 transport activity. This provides an explanation for the phenotype observed in this dog breed. Additionally, we showed that N333 is involved in the affinity of Glut9 for urate when mutated in serine, suggesting that N333 locates in the binding pocket. Finally, we showed that a frequent SNP of Glut9, V253I, displays low transport activity due to defective targeting. This is suggestive of a protective effect against hyperuricemia and gout in humans.

## Author Contributions

AR, LS, and OB designed the study and analyzed the data. AR and IG realized the experiments. AR and OB wrote the manuscript.

## Conflict of Interest Statement

The authors declare that the research was conducted in the absence of any commercial or financial relationships that could be construed as a potential conflict of interest.

## References

[B1] Anzai N. Ichida K. Jutabha P. Kimura T. Babu E. Jin C. J. (2008). Plasma urate level is directly regulated by a voltage-driven urate efflux transporter Uratv1 (*Slc2A9*) in humans. *J. Biol. Chem.* 283 26834–26838. 10.1074/jbc.C800156200 18701466

[B2] Bannasch D. Safra N. Young A. Karmi N. Schaible R. S. Ling G. V. (2008). Mutations in the SLC2A9 gene cause hyperuricosuria and hyperuricemia in the dog. *PLoS Genet.* 4:e1000246. 10.1371/journal.pgen.1000246 18989453 PMC2573870

[B3] Bibert S. Hess S. K. Firsov D. Thorens B. Geering K. Horisberger J. D. (2009). Mouse GLUT9: evidences for a urate uniporter. *Am. J. Physiol. Renal Physiol.* 297 F612–F619. 10.1152/ajprenal.00139.2009 19587147

[B4] Bonny O. Chraibi A. Loffing J. Jaeger N. F. Grunder S. Horisberger J. D. (1999). Functional expression of a pseudohypoaldosteronism type I mutated epithelial Na+ channel lacking the pore-forming region of its alpha subunit. *J. Clin. Invest.* 104 967–974. 10.1172/JCI6821 10510337 PMC408554

[B5] Braun P. von Heijne G. (1999). The aromatic residues Trp and Phe have different effects on the positioning of a transmembrane helix in the microsomal membrane. *Biochemistry* 38 9778–9782. 10.1021/bi990923a 10423258

[B6] Caulfield M. J. Munroe P. B. O’neill D. Witkowska K. Charchar F. J. Doblado M. (2008). SLC2A9 is a high-capacity urate transporter in humans. *PLoS Med.* 5:e197. 10.1371/journal.pmed.0050197 18842065 PMC2561076

[B7] Clemencon B. Luscher B. P. Fine M. Baumann M. U. Surbek D. V. Bonny O. (2014). Expression, purification, and structural insights for the human uric acid transporter, GLUT9, using the *Xenopus laevis* oocytes system. *PLoS One* 9:e108852. 10.1371/journal.pone.0108852 25286413 PMC4186817

[B8] Dehghan A. Kottgen A. Yang Q. Hwang S. J. Kao W. L. Rivadeneira F. (2008). Association of three genetic loci with uric acid concentration and risk of gout: a genome-wide association study. *Lancet* 372 1953–1961. 10.1016/S0140-6736(08)61343-4 18834626 PMC2803340

[B9] Deng D. Xu C. Sun P. Wu J. Yan C. Hu M. (2014). Crystal structure of the human glucose transporter GLUT1. *Nature* 510 121–125. 10.1038/nature13306 24847886

[B10] Dinour D. Gray N. K. Campbell S. Shu X. Sawyer L. Richardson W. (2010). Homozygous SLC2A9 mutations cause severe renal hypouricemia. *J. Am. Soc. Nephrol.* 21 64–72. 10.1681/ASN.2009040406 19926891 PMC2799278

[B11] Dinour D. Gray N. K. Ganon L. Knox A. J. Shalev H. Sela B. A. (2012). Two novel homozygous SLC2A9 mutations cause renal hypouricemia type 2. *Nephrol. Dial. Transplant.* 27 1035–1041. 10.1093/ndt/gfr419 21810765

[B12] Geering K. Beggah A. Good P. Girardet S. Roy S. Schaer D. (1996). Oligomerization and maturation of Na,K-ATPase: functional interaction of the cytoplasmic NH2 terminus of the beta subunit with the alpha subunit. *J. Cell Biol.* 133 1193–1204. 10.1083/jcb.133.6.1193 8682858 PMC2120891

[B13] Harris M. Firsov D. Vuagniaux G. Stutts M. J. Rossier B. C. (2007). A novel neutrophil elastase inhibitor prevents elastase activation and surface cleavage of the epithelial sodium channel expressed in *Xenopus laevis* oocytes. *J. Biol. Chem.* 282 58–64. 10.1074/jbc.M605125200 17090546

[B14] Hollis-Moffatt J. E. Gow P. J. Harrison A. A. Highton J. Jones P. B. Stamp L. K. (2011). The *SLC2A9* nonsynonymous Arg265His variant and gout: evidence for a population-specific effect on severity. *Arthritis Res. Ther.* 13:R85. 10.1186/ar3356 21658257 PMC3218899

[B15] Hurba O. Mancikova A. Krylov V. Pavlikova M. Pavelka K. Stiburkova B. (2014). Complex analysis of urate transporters SLC2A9, SLC22A12 and functional characterization of non-synonymous allelic variants of GLUT9 in the Czech population: no evidence of effect on hyperuricemia and gout. *PLoS One* 9:e107902. 10.1371/journal.pone.0107902 25268603 PMC4182324

[B16] Jeannin G. Chiarelli N. Gaggiotti M. Ritelli M. Maiorca P. Quinzani S. (2014). Recurrent exercise-induced acute renal failure in a young Pakistani man with severe renal hypouricemia and SLC2A9 compound heterozygosity. *BMC Med. Genet.* 15:3. 10.1186/1471-2350-15-3 24397858 PMC3890613

[B17] Keilin J. (1959). The biological significance of uric acid and guanine excretion. *Biol. Rev. Camb. Philos. Soc.* 34 265–296. 10.1111/j.1469-185X.1959.tb01447.x 18498612

[B18] Li S. Sanna S. Maschio A. Busonero F. Usala G. Mulas A. (2007). The GLUT9 gene is associated with serum uric acid levels in Sardinia and Chianti cohorts. *PLoS Genet.* 3:e194. 10.1371/journal.pgen.0030194 17997608 PMC2065883

[B19] Long W. Panigrahi R. Panwar P. Wong K. O’Neill D. Chen X. Z. (2017). Identification of key residues for urate specific transport in human glucose transporter 9 (hSLC2A9). *Sci. Rep.* 7:41167. 10.1038/srep41167 28117388 PMC5259734

[B20] Long W. Panwar P. Witkowska K. Wong K. O’neill D. Chen X. Z. (2015). Critical roles of two hydrophobic residues within human glucose transporter 9 (hslc2A9) in substrate selectivity and urate transport. *J. Biol. Chem.* 290 15292–15303. 10.1074/jbc.M114.611178 25922070 PMC4463468

[B21] Mancikova A. Krylov V. Hurba O. Sebesta I. Nakamura M. Ichida K. (2016). Functional analysis of novel allelic variants in URAT1 and GLUT9 causing renal hypouricemia type 1 and 2. *Clin. Exp. Nephrol.* 20 578–584. 10.1007/s10157-015-1186-z 26500098

[B22] Matsuo H. Chiba T. Nagamori S. Nakayama A. Domoto H. Phetdee K. (2008). Mutations in glucose transporter 9 gene SLC2A9 cause renal hypouricemia. *Am. J. Hum. Genet.* 83 744–751. 10.1016/j.ajhg.2008.11.001 19026395 PMC2668068

[B23] McArdle P. F. Parsa A. Chang Y. P. Weir M. R. O’connell J. R. Mitchell B. D. (2008). Association of a common nonsynonymous variant in GLUT9 with serum uric acid levels in old order amish. *Arthritis Rheum.* 58 2874–2881. 10.1002/art.23752 18759275 PMC2779583

[B24] Mueckler M. Caruso C. Baldwin S. A. Panico M. Blench I. Morris H. R. (1985). Sequence and structure of a human glucose transporter. *Science* 229 941–945. 10.1126/science.3839598 3839598

[B25] Mueckler M. Makepeace C. (2002). Analysis of transmembrane segment 10 of the Glut1 glucose transporter by cysteine-scanning mutagenesis and substituted cysteine accessibility. *J. Biol. Chem.* 277 3498–3503. 10.1074/jbc.M109157200 11713254

[B26] Nomura N. Verdon G. Kang H. J. Shimamura T. Nomura Y. Sonoda Y. (2015). Structure and mechanism of the mammalian fructose transporter GLUT5. *Nature* 526 397–401. 10.1038/nature14909 26416735 PMC4618315

[B27] Pascual J. M. Wang D. Lecumberri B. Yang H. Mao X. Yang R. (2004). GLUT1 deficiency and other glucose transporter diseases. *Eur. J. Endocrinol.* 150 627–633. 10.1530/eje.0.1500627 15132717

[B28] Pettersen E. F. Goddard T. D. Huang C. C. Couch G. S. Greenblatt D. M. Meng E. C. (2004). UCSF Chimera–a visualization system for exploratory research and analysis. *J. Comput. Chem.* 25 1605–1612. 10.1002/jcc.20084 15264254

[B29] Polasek O. Gunjaca G. Kolcic I. Zgaga L. Dzijan S. Smolic R. (2010). Association of nephrolithiasis and gene for glucose transporter type 9 (SLC2A9): study of 145 patients. *Croat. Med. J.* 51 48–53. 10.3325/cmj.2010.51.48 20162745 PMC2829176

[B30] Preitner F. Bonny O. Laverriere A. Rotman S. Firsov D. Da Costa A. (2009). Glut9 is a major regulator of urate homeostasis and its genetic inactivation induces hyperuricosuria and urate nephropathy. *Proc. Natl. Acad. Sci. U.S.A.* 106 15501–15506. 10.1073/pnas.0904411106 19706426 PMC2741280

[B31] Roch-Ramel F. Guisan B. (1999). Renal transport of urate in humans. *News Physiol. Sci.* 14 80–84. 10.1152/physiologyonline.1999.14.2.80 11390825

[B32] Sorensen L. B. (1965). Role of the intestinal tract in the elimination of uric acid. *Arthritis Rheum.* 8 694–706. 10.1002/art.1780080429 5859543

[B33] Stiburkova B. Taylor J. Marinaki A. M. Sebesta I. (2012). Acute kidney injury in two children caused by renal hypouricaemia type 2. *Pediatr. Nephrol.* 27 1411–1415. 10.1007/s00467-012-2174-0 22527535

[B34] Sulem P. Gudbjartsson D. F. Walters G. B. Helgadottir H. T. Helgason A. Gudjonsson S. A. (2011). Identification of low-frequency variants associated with gout and serum uric acid levels. *Nat. Genet.* 43 1127–1130. 10.1038/ng.972ng 21983786

[B35] Sun L. Zeng X. Yan C. Sun X. Gong X. Rao Y. (2012). Crystal structure of a bacterial homologue of glucose transporters GLUT1-4. *Nature* 490 361–366. 10.1038/nature11524 23075985

[B36] Vitart V. Rudan I. Hayward C. Gray N. K. Floyd J. Palmer C. N. (2008). SLC2A9 is a newly identified urate transporter influencing serum urate concentration, urate excretion and gout. *Nat. Genet.* 40 437–442. 10.1038/ng.106 18327257

[B37] Watts R. W. (1966). Uric acid production with particular reference to the role of xanthine oxidase and its inhibition. *Proc. R. Soc. Med.* 59 287–292. 5937675 10.1177/003591576605900401 PMC1900614

[B38] Webb B. Sali A. (2014). Comparative protein structure modeling using MODELLER. *Curr. Protoc. Bioinformatics* 47 5.6.1–5.6.32. 10.1002/0471250953.bi0506s47 25199792

[B39] Wu X. W. Muzny D. M. Lee C. C. Caskey C. T. (1992). Two independent mutational events in the loss of urate oxidase during hominoid evolution. *J. Mol. Evol.* 34 78–84. 10.1007/BF00163854 1556746

[B40] Yang Q. Kottgen A. Dehghan A. Smith A. V. Glazer N. L. Chen M. H. (2010). Multiple genetic loci influence serum urate levels and their relationship with gout and cardiovascular disease risk factors. *Circ. Cardiovasc. Genet.* 3 523–530. 10.1161/CIRCGENETICS.109.934455 20884846 PMC3371395

